# Longitudinal study on blood and biochemical indexes of Tibetan and Han in high altitude area

**DOI:** 10.3389/fpubh.2023.1282051

**Published:** 2023-11-14

**Authors:** ZhiMin Yuan, YuanWu Zou, XiaoXing Liu, LongHao Wang, Cheng Chen

**Affiliations:** ^1^Department of Clinical Laboratory, Shaanxi Provincial Cancer Hospital Affiliated to Xi'an Jiaotong University, Xi'an, China; ^2^Department of Clinical Laboratory, Ali District People's Hospital, Tibet Ali, China; ^3^Department of Clinical Laboratory, Tuberculosis Prevent and Care Hospital of Shanxi Province, Xi’an, China; ^4^Department of Otolaryngology and Neck Surgery, Shanghai Ninth People’s Hospital, Shanghai Jiaotong University, Shanghai, China; ^5^Key Laboratory of Shaanxi Province for Craniofacial Precision Medicine Research, College of Stomatology, Xi'an Jiaotong University, Xi'an, China

**Keywords:** high altitude, blood biochemical indexes, longitudinal analysis, inhabitant, Tibetan

## Abstract

**Objective:**

This study aims to review the blood routine and biochemical indicators of the plateau population for three consecutive years, and analyze the impact of the plateau on these blood indicators of the Tibetan population and the Han immigrant population.

**Method:**

These parameters were extracted from the Laboratory Department of Ali District People’s Hospital in Tibet from January 2019 to December 2021, including blood routine, liver and kidney function, blood lipids, myocardial enzyme spectrum, and rheumatic factor indicators. Changes in these parameters were analyzed over 3 consecutive years according to inclusion and exclusion criteria.

**Result:**

A total of 114 Tibetans and 93 Hans participated in the study. These parameters were significantly different between Tibetan and Han populations. Red blood cells (RBC), hemoglobin (HGB), hematocrit (HCT), mean hemoglobin content (MCH), mean corpuscular hemoglobin concentration (MCHC), white blood cells (WBC), lymphocytes (LYMPH) and monocytes (MONO) were significantly higher in Hans than Tibetans (*p* < 0.05). Biochemically, total bilirubin (TBIL), direct bilirubin (DBIL), albumin (ALB), urea nitrogen (Urea), creatinine (Cr), uric acid (UA), glucose (GLU), triglycerides (TG) and creatine kinase isoenzyme (CKMB) were significantly higher in Hans than Tibetans; aspartate aminotransferase (AST), glutamyl transpeptidase (GGT), alkaline phosphatase (ALP), antistreptolysin (ASO), and C-reactive protein (CRP) were significantly higher in Tibetans than Hans (*p* < 0.05). There were no obvious continuous upward or downward trend of the parameters for 3 consecutive years.

**Conclusion:**

In high-altitude areas, Han immigrants have long-term stress changes compared with Tibetans. The main differences are reflected in the blood system, liver and kidney functions, etc., which provide basic data for further research on the health status of plateau populations.

## Introduction

Above 3,500 meters is defined as extremely high. People living or traveling at high altitudes experience severe cold, hypothermia, lack of oxygen, radiation and drought (1–3). Generations of people living at high altitudes may have adapted their bodies to the environment (4–8), and their metabolism may be different from that of sojourners (9, 10). When people originally living at low altitudes migrate to higher altitudes, they are often unable to adapt to the local environment (11), and usually have acute altitude sickness, high altitude lung disease, high altitude encephalopathy, etc. (12, 13).

Most studies have dealt with physiological and genetic changes in general populations or athletes following migration from low to high altitudes (14). Other studies have compared physiological changes in people who have lived at high altitudes for generations, such as Tibetans, Andeans, and Ethiopians (4, 15). Few studies have been conducted on populations that have adapted to low altitudes and resettled at high altitudes (9, 16). Adaptation of the body is a long process, requiring thousands of years of genetic integration for slow changes to occur. The effects of environmental change may be more pronounced for people who have adapted to lower altitudes and settled at higher altitudes. Studying their pathophysiological changes may help to explore the impact of the environment on the human body itself. For example, changes in the partial pressure of oxygen may alter how quickly the bone marrow produces hemoglobin, but the long-term effects are still unknown.

The average altitude of the Ngari area in Tibet is 4,300 meters, which is one of the high-altitude areas inhabited by humans (17). Over the past 30 years of China’s reform and development, a large number of ethnic groups represented by the Han nationality have settled in Ngari, Tibet. Longitudinal observations of metabolic and physiological changes in their bodies as they acclimate to altitude will help us understand the true impact of high altitude on these individuals.

Hematological indicators are commonly used indicators to observe the health status of the body and abnormal metabolism (18). The reference range varies with altitude. Several cross-sectional studies have shown that the hemoglobin of indigenous high-altitude Tibetan populations is significantly lower than that of settled high-altitude Han populations (19). Some studies have also found that metabolic abnormalities in high-altitude populations, such as hyperuricemia, are associated with metabolic diseases (20–22). A cross-sectional study found that the overall prevalence of metabolic syndrome at high altitudes in China was 3.6% (5.9% in men and 1.8% in women). Obesity and hypertension are more common than dyslipidemia and hyperglycemia in adults at high altitudes. Residents at very high altitudes (≥3,000 m) have a reduced risk of obesity and dyslipidemia and an increased risk of hypertension (23). Therefore, in this study, the serological and blood metabolic indicators of long-term settled and native populations were continuously observed for 3 years to observe the impact of high altitude on long-term settled non-high-altitude lineage populations. In addition, the study also analyzed the correlation of baseline data with blood routine and biochemical indicators in native and settled populations to understand the health status, physiological and metabolic differences of high-altitude populations.

## Materials and methods

### Materials

In this study, long-term residents who underwent continuous physical examination in the clinical laboratory of Ali District People’s Hospital (altitude of 4,300 meters) from January 2019 to December 2021 were selected. Inclusion criteria were: (1) long-term residence in high-altitude areas for at least 5 years; (2) the age distribution was 18–60 years old. Exclusion criteria in this study were based on health industry standards, and reference was made to authoritative books and literature. It is based on the opinions of laboratory and clinical experts as well as the results of statistical analysis. It is listed below: (1) Serological indicators (GLU ≥ 7.0 mmol/L; WBC < 3.0 × 10^9^/L or >15.0 × 10^9^/L; HGB < 90 g/L; ALB < 25 g/L; ALT > 80 U/L and AST > 80 U/L). (2) Congenital immune system and hematological diseases; (3) pregnant women; (4) Over 60 years old. This study was approved by the Ethics Committee of Ali District People’s Hospital (No. 2022004). All methods were carried out in accordance with relevant guidelines and regulations.

### Blood and serological test methods

In this study, all physical examination subjects adopted uniform blood collection equipment and hematological testing methods. The steps are as follows: 2 and 3 ml blood were collected from veins in an anticoagulant vacuum tube (EDTA-K2) and heparin procoagulant tube. The 2 ml blood were analyzed by Seisen Micron XT-4000i from Japan Seisen Micron Co., LTD, and 3 ml blood were analyzed by Hitachi 7,180 from Japan Hitachi Co., LTD. After blood was collected, the test was completed on the machine on site, and the longest time for blood samples was no more than 30 min. During the test, quality control was carried out with the supporting quality control materials of the instruments. The reference interval standard for all routine indexes was in accordance with the Health industry standard WS/T 404, 405-2012 of the People’s Republic of China.

### Collection of hematological data

The hematological data were collected for 3 consecutive years. Red blood cell related hematological indicators include erythrocyte (RBC), hemoglobin (HGB), hematocrit (HCT) and mean corpuscular volume (MCV), mean hemoglobin content (MCH), mean erythrocyte hemoglobin concentration (MCHC), red blood cell distribution width (RDW) and standard deviation of red blood cell distribution width (RDW-SD). Leukocyte related hematologic indicators include white blood cell (WBC), neutrophil (NEUT), lymphocyte (LYMPH), monocyte (MONO), eosinophil (EO), basophil (BASO), and their percent, immature granulocyte ratio (IG%). Platelet-related hematologic indicators include platelet (PLT), platelet distribution width (PDW), mean platelet volume (MPV), thrombocytocrit (PCT) and platelet-large cell rate (P-LCR).

### Collection of biochemical data of hematology

Blood biochemical data were collected for 3 consecutive years. Indicators related to liver function include alanine transaminase (ALT), aspertate aminotransferase (AST), total bilirubin (TBIL), direct bilirubin (DBIL), alkaline phosphatase (ALP), glutamyl transpeptidase (GGT), total protein (TP), and albumin (ALB). Renal function related indicators include urea nitrogen (Urea), creatinine (Cr), uric acid (UA), and glucose (GLU). Blood lipid related hematologic indicators include cholesterol (CHOL), triglyceride (TG), high-density lipoprotein (HDL), and low-density lipoprotein (LDL). Myocardial enzymology related hematological indicators include creatine phosphate kinase (CK), lactate dehydrogenase (LDH), hydroxybutyrate dehydrogenase (HBDH), and creatine kinase isoenzyme (CKMB). Serological indicators associated with rheumatism include antistreptolysin (ASO), rheumatoid factors (RF), C-reactive protein (CRP).

### Statistical method

Statistical analysis was performed using Statistical Product and Service Solutions (SPSS) 23.0 software. Categorical variables were described as proportions and compared using the *χ*^2^ test. The measurement data are described by means ± standard deviation (X ± SD). Analysis of variance was used for comparison between groups, and paired Student’s *t*-test was used for comparison within groups. *p* < 0.05 was considered statistically significant.

## Results

### General information analysis

This study collected a total of 207 samples of people who met the conditions and lived in extremely high altitude areas for more than 5 years, including 114 Tibetans and 93 Hans. The Tibetan were locality people (altitude of 4,300 meters) and Han population were all from no high-altitude areas (altitude of 200–300 meters). Table 1 presents baseline data, including demographics (age and gender), BMI, blood pressure (diastolic pressure and systolic pressure), heart rate, smoking, drink and history of disease characteristics.

**Table 1:** Comparison of age and gender in the Tibetan-Han group.

Parameter	Cases (*n* = 207)	Tibetan (*n* = 114)	Han (*n* = 93)	*χ* ^2^	*p*-value
Age (mean ± SD), years	32.7 ± 3.5	33.6 ± 6.8	31.5 ± 7.3	0.143	0.706
Gender					
male	144 (70%)	65 (57%)	79 (85%)	18.87	<0.001
female	63 (30%)	49 (43%)	14 (15%)		

Data are presented as the means ± standard deviation or percent. *p* values were determined by Student’s *t*-test for continuous variables and the chi-square test for categorical variables.

### Changes and comparison of RBC related indexes in high altitude area

**Figure 1:**
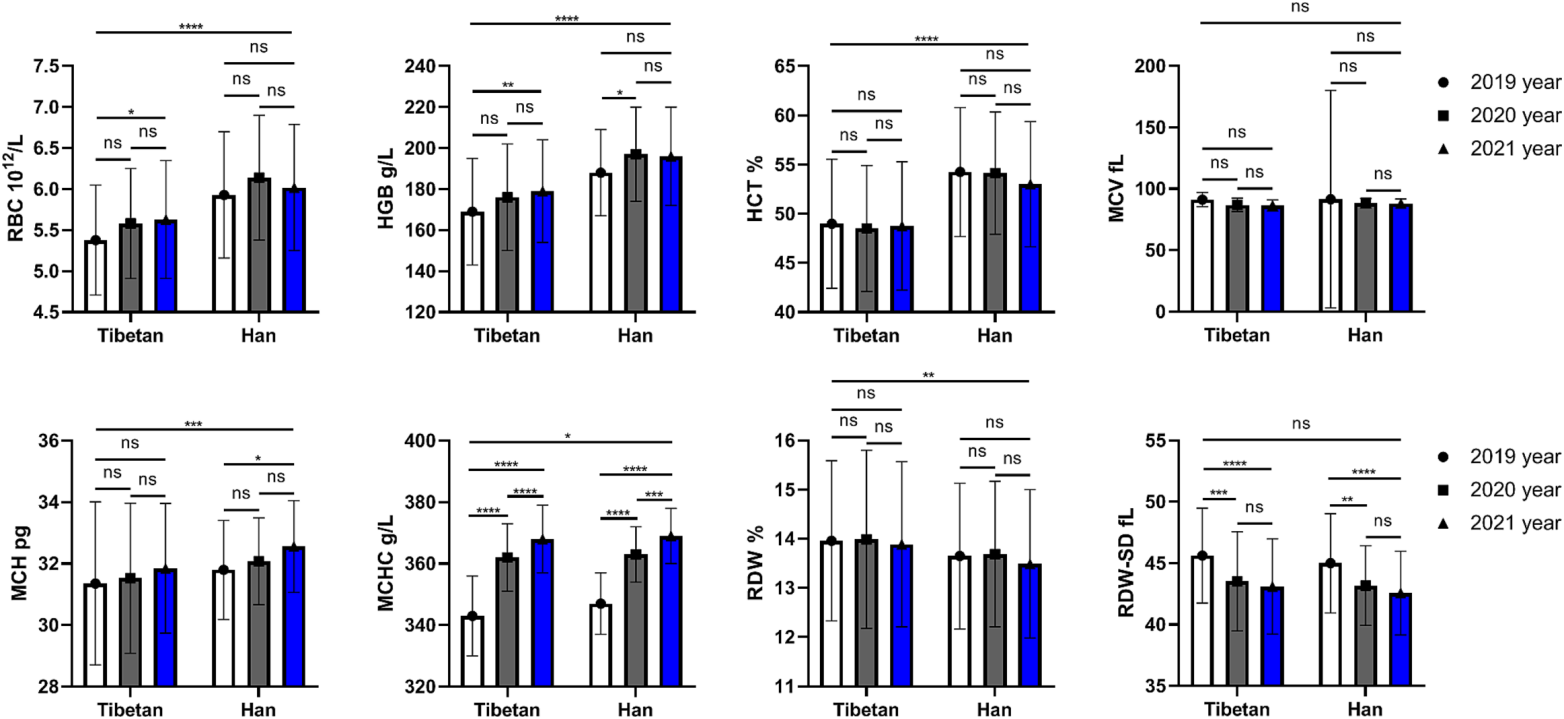
Comparison of RBC related indexes in Tibetan and Han. Reference ranges: RBC: 3.99–5.70 10^12^/L, HGB: 125-182 g/L, HCT: 38.19–57.91%, MCV: 81.8–95.5 fL, MCH: 27.0–32.3 pg., MCHC: 324–350 g/L, RDW-SD: 37.1–45.7 fL, RDW: 12.0–13.6%. *p < 0.05, **p < 0.01, ***p < 0.001, Analysis of variance was used for comparison between Tibetan and Han, and paired Student’s *t*-test was used for comparison within groups. *p* <  0.05 was considered statistically significant.

As shown in Figure 1, compared with Tibetans, RBC, HGB, HCT, MCH (*F* = 73.31, 93.30, 93.98, 11.69, *p* < 0.001) and MCHC (*F* = 5.358, *p* = 0.021) values in blood of Han people living in high-altitude areas for three consecutive years increased significantly, while the values of RDW (*F* = 6.685, *p* = 0.01) downgraded significantly. There were statistically significant differences in RBC (*F* = 4.701, *p* = 0.009), HGB (*F* = 8.385, *p* = 0.0003), MCH (*F* = 4.865, *p* = 0.008), MCHC (*F* = 266.3, *p* < 0.001) and RDW-SD (*F* = 24.36, *p* < 0.001) among groups in 3 consecutive years.

However, there is no significant trend and difference in the comparison of each year within each nationality. We found that compared with 2019, RBC and HGB values in Tibetan people’s blood increased significantly in 2021, and the difference was statistically significant (*t* = 3.687, *p* = 0.025, *t* = 4.379, *p* = 0.006). Compared with 2019, HGB value in blood of Han population increased significantly in 2020, with statistical significance (*t* = 3.560, *p* = 0.032). Comparison between different years showed that values of MCHC in blood gradually increased, and there were statistical differences between Tibetan population (2019 vs. 2020, 2020 vs. 2021, and 2019 vs. 2021, *t* = 18.94, 5.981, 24.92, *p* < 0.001) and Han population (2019 vs. 2020, 2020 vs. 2021, and 2019 vs. 2021, *t* = 14.41, 5.402, 19.81, *p* < 0.001). Compared with 2019, the values of MCH in blood of Han population reduced significantly in 2021, with statistical significance (*t* = 3.568, *p* = 0.032). The value of RDW-SD in blood decreased year by year, and the test results were similar in Tibetan and Han populations, which were significantly higher in 2019 than in the following 2 years, and the difference was statistically significant (Tibetan: 2019 vs. 2020 and 2019 vs. 2021, *t* = 5.905, 7.092, *p* < 0.001; Han: 2019 vs. 2020 and 2019 vs. 2021, *t* = 4.646, 6.201, *p* < 0.001).

### Changes and comparative of WBC related indexes in high altitude area

**Figure 2:**
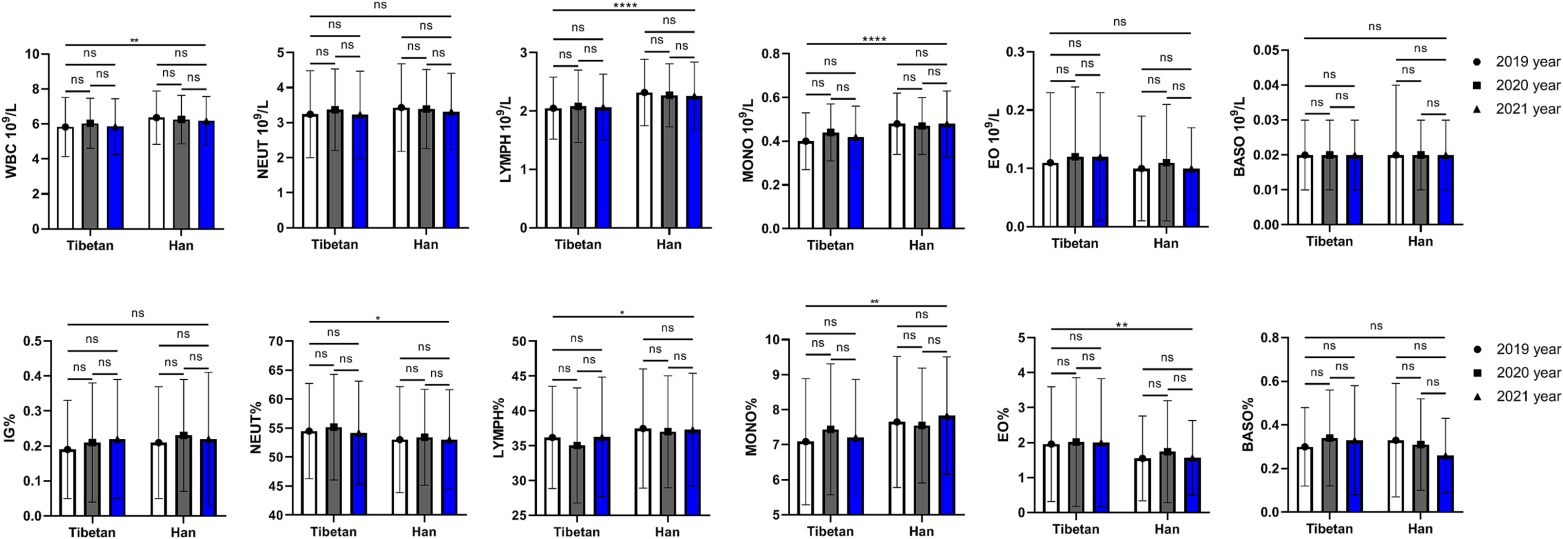
Comparison of WBC related indexes in Tibetan and Han. Reference ranges: WBC: 4.00–10.00 10^9^/L, NEUT#: 1.80–6.98 10^9^/L, YMPH#: 1.26–3.35 10^9^/L, MONO#: 0.29–0.95 10^9^/, EO#: 0.03–0.59 10^9^/L, BASO#: 0.01–0.07 10^9^/L, NEUT%: 41.0–70.7%, LYMPH%: 19.1–47.9%, MONO%: 5.2–15.2%, EO%: 0.6–7.6%, BASO%: 0.1–1.2%, IG%: 0–0.5%. *p < 0.05, **p < 0.01, ***p < 0.001. Analysis of variance was used for comparison between Tibetan and Han, and paired Student’s *t*-test was used for comparison within groups. *p* <  0.05 was considered statistically significant.

As shown in Figure 2, compared with Tibetans, the values of LYMPH and MONO in blood of Han population increased (*F* = 22.37, 26.47, *p* < 0.001), while there were no significant differences in that of NEUT, EO and BASO. The ratios of leukocyte lineages in blood of Tibetan and Han groups were different, and the values of NEUT% (*F* = 4.275, *p* = 0.039), LYMPH% (*F* = 4.715, *p* = 0.030), MONO% (*F* = 9.189, *p* = 0.003) and EO% (*F* = 8.786, *p* = 0.003) were significantly different. The total WBC counts showed significant abnormalities over time between Tibetan and Han (*F* = 8.540, *p* = 0.004), while values of IG% did not show significant abnormalities (*F* = 1.001, *p* = 0.317). The results showed that there was no significant and trend in the changes of white blood cell lines over time within ethnic groups.

### Changes and comparative of PLT related indexes in high altitude area

**Figure 3:**
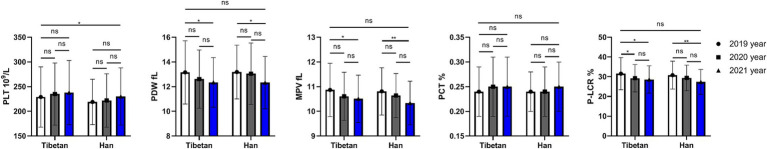
Comparison of PLT related indexes in Tibetan and Han. Reference ranges: PLT: 100–330 10^9^/L, PDW: 10.1–16.1 fL, MPV: 9.3–12.1 fL, P-LCR: 18.5–42.3%, PCT: 0.17–0.32%. *p < 0.05, **p < 0.01, ***p < 0.001. Analysis of variance was used for comparison between Tibetan and Han, and paired Student’s *t*-test was used for comparison within groups. *p* <  0.05 was considered statistically significant.

As shown in Figure 3, compared with Tibetans, values of PLT decreased significantly for 3 consecutive years, and the difference was statistically significant (*F* = 4.762, *p* = 0.030), while the values of PDW, MPV, and P-LCR gradually decreased over time, but there was no significant difference between Tibetan and Han. There were statistically significant annual differences in PDW (*F* = 6.840, *p* = 0.001), MPV (*F* = 9.502, *p* < 0.001), P-LCR (*F* = 10.79, *p* < 0.001). The PDW and MPV values of Tibetan and Han populations in 2021 were significantly lower than those in 2019, and the differences were statistically significant (PDW: Tibetan 2019 vs. 2021, *t* = 3.764, *p* = 0.022, Han 2019 vs. 2021, *t* = 3.609, *p* = 0.029; MPV: Tibetan 2019 vs. 2021, *t* = 3.988, *p* = 0.014 Han 2019 vs. 2021, *t* = 4.703, *p* = 0.029). The value of P-LCR in blood decreased year by year, which were significantly higher in 2019 than in the following 2 years, and the difference was statistically significant in Tibetans (2019 vs. 2021, *t* = 4.493, *p* = 0.004). Compared with 2019, the values of P-LCR in blood of Han population reduced significantly in 2021, with statistical significance (*t* = 4.755, *p* = 0.002).

### Trends and comparative analysis of liver function indexes in blood biochemistry in high altitude area

**Figure 4:**
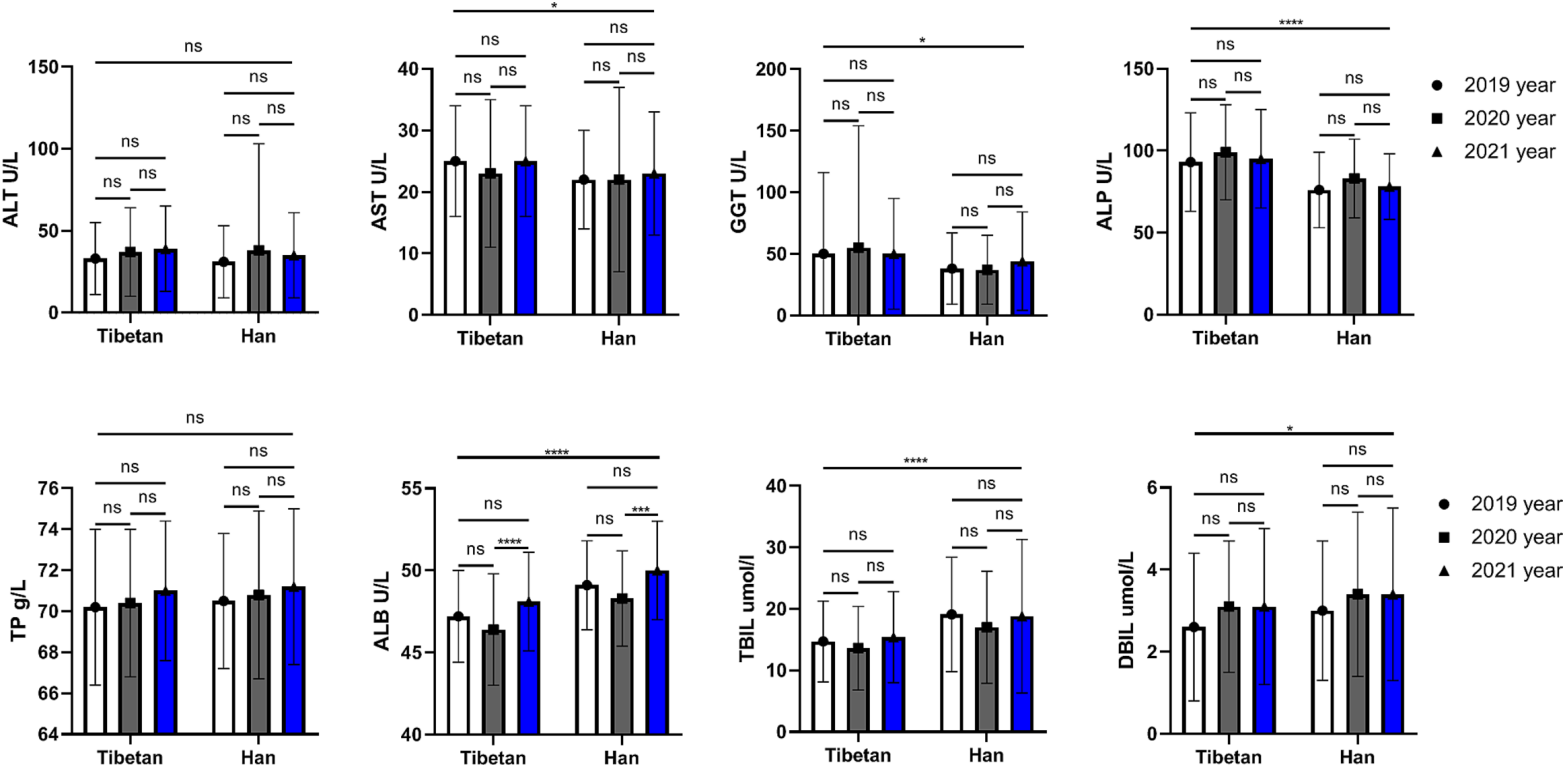
Comparison of liver function related indexes in Tibetan and Han. Reference ranges: ALT: 5–40 U/L, AST: 5–40 U/L, GGT: 5–50 U/L, ALP: 35–142 U/L, TP: 65.0–85.0 g/L, ALB: 35.0–54.0 g/L, TBIL: 3.5–20.5umol/L. DBIL: 0–6.8umol/L. *p < 0.05, **p < 0.01, ***p < 0.001. Analysis of variance was used for comparison between Tibetan and Han, and paired Student’s *t*-test was used for comparison within groups. *p* <  0.05 was considered statistically significant.

As shown in Figure 4, the AST (*F* = 5.372, *p* = 0.021), GGT (*F* = 6.403, *p* = 0.012), and ALP (*F* = 60.08, *p* < 0.001) values of Han people in the same high altitude area were significantly lower than those of Tibetan people, while the ALB, TBIL (*F* = 62.23, 28.46, *p* < 0.001) and DBIL (*F* = 4.993, *p* = 0.026) values were significantly higher than those of Tibetan people. Paired test analysis found that the blood ALB value in 2020 was significantly higher than that in 2021 both in Tibetan and Han people (*t* = 6.080, 5.491, *p* < 0.001).

### Trends and comparative analysis of renal function related indexes in blood biochemistry in high altitude area

**Figure 5:**
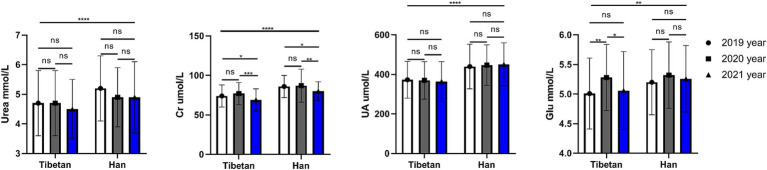
Comparison of renal function related indexes in Tibetan and Han. Reference ranges: Urea: 2.9–8.2 mmol/L, Cr: 45-104umol/L, UA: 142–416μmol/L, GLU: 3.89–6.11 mmol/L. *p < 0.05, **p < 0.01, ***p < 0.001. Analysis of variance was used for comparison between Tibetan and Han, and paired Student’s *t*-test was used for comparison within groups. *p* <  0.05 was considered statistically significant.

As shown in Figure 5, compared with Tibetan population, Urea, Cr and UA (*F* = 20.66, 82.63, 87.63, *p* < 0.001) of blood renal function indexes in Han population increased significantly for 3 consecutive years, and the difference was statistically significant. A 3-year paired comparative analysis showed that there was no difference in UA and Urea in Tibetan or Han nationality, and there was no obvious upward or downward trend. The value of Cr in blood decreased year by year, and the test results were similar in Tibetan and Han populations, which were significantly lower in 2021 than in the last 2 years, and the difference was statistically significant (Tibetan: 2020 vs. 2021, *t* = 5.696, *p* < 0.001, 2019 vs. 2021, *t* = 3.560, *p* = 0.032; Han: 2020 vs. 2021, *t* = 4.502, *p* = 0.004, 2019 vs. 2021, *t* = 3.859, *p* = 0.018).

The blood GLU index of Han people in high altitude area was significantly higher than that of Tibetans for three consecutive years (*F* = 9.206, *p* = 0.003). Paired test found that the blood GLU of Tibetans was unstable for three consecutive years, and the blood GLU in 2020 was significantly higher than that in 2019 and 2021 (Tibetan: 2019 vs. 2020, *t* = 4.923, *p* = 0.002, 2020 vs. 2021, *t* = 4.011, *p* = 0.013), while the blood GLU of Han Chinese was not significantly changed for three consecutive years, and no statistical difference was found by comparison.

### Trends and comparative analysis of lipid related indexes in blood biochemistry in high altitude area

**Figure 6:**
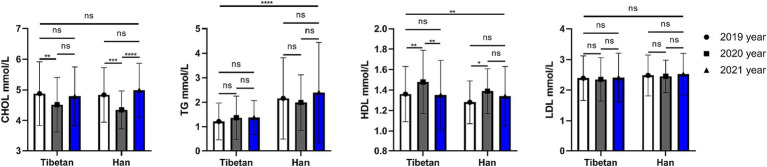
Comparison of lipid related indexes in Tibetan and Han. Reference ranges: CHOL: 2.83–5.17 mmol/L, TG: 0.34–1.70 mmol/, HDL-C: 1.16–1.55 mmol/L, LDL-C: 0–3.37 mmol/L. *p < 0.05, **p < 0.01, ***p < 0.001. Analysis of variance was used for comparison between Tibetan and Han, and paired Student’s *t*-test was used for comparison within groups. *p* <  0.05 was considered statistically significant.

As shown in the Figure 6, there is no significant difference in blood CHOL values between Han and Tibetan at the same high sea altitude. Compared with the Tibetans, the blood CHOL value of the Han people in the same high sea altitude area has no significant difference, while the TG value is significantly increased (*F* = 72.90, *p* < 0.001). Tibetans have significantly higher levels of HDL (*F* = 7.028, *p* = 0.008) in their blood than Han people, while LDL levels are almost the same. The HDL level of Tibetans in 2020 was significantly higher than that in 2019 and 2021 (2019 vs. 2020, *t* = 4.567, *p* = 0.004, 2020 vs. 2021, *t* = 4.947, *p* = 0.002). The HDL content of Han Chinese in 2021 was significantly higher than that in 2019 (2019 vs. 2020, *t* = 3.781, *p* = 0.021).

### Trends and comparative analysis of myocardial enzymes in blood biochemistry in high altitude area

**Figure 7:**
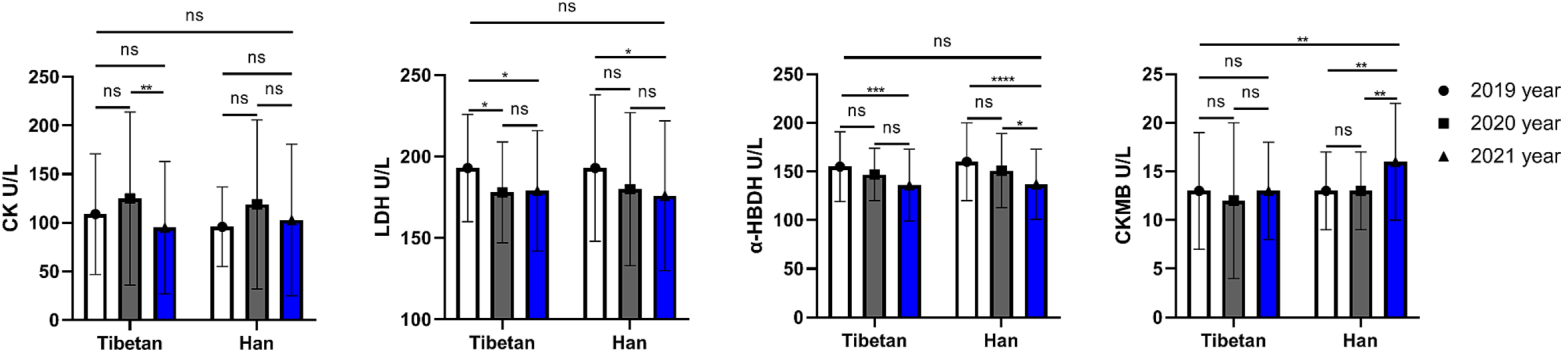
Comparison of myocardial enzymes related indexes in Tibetan and Han. Reference ranges: CK: 38–174 U/L, LDH: 109–245 U/L, α-HBDH: 72–182 U/L, CKMB: 0-24 U/L. *p < 0.05, **p < 0.01, ***p < 0.001. Analysis of variance was used for comparison between Tibetan and Han, and paired Student’s *t*-test was used for comparison within groups. *p* <  0.05 was considered statistically significant.

As shown in the Figure 7, compared with Tibetans, CKMB was higher in Han, and the difference was statistically significant (*F* = 8.243, *p* = 0.004). Pairings between years also found differences (*F* = 6.697, *p* = 0.001). The change of CKMB in Tibetans was not significant in three consecutive years, but it was significantly increased in Han population in the last 2 years (2020 vs. 2021 and 2019 vs. 2021, *t* = 5.026, *p* = 0.001). CK, LDH, and α-HBDH were not significantly different between Tibetan and Han populations, but were different in 3 years of continuous testing. There was no significant change in CK value in Han population, but it was significantly higher in Tibetan population in 2020 than in 2021 (*t* = 4.398, *p* = 0.006). The LDH value decreased gradually for three consecutive years, and the LDH value decreased significantly in 2021 compared with 2019, regardless of Tibetan or Han nationality, and the difference was statistically significant (Tibetan: *t* = 3.763, *p* = 0.022, Han: *t* = 4.127, *p* = 0.01). Similarly, the α-HBDH value also decreased gradually for 3 consecutive years. For Tibetan and Han people, the α-HBDH value decreased significantly in 2021 compared with 2019, and the difference was statistically significant (*t* = 5.686, 6.217, *p* < 0.001).

### Trends and comparative analysis of hematologic indexes related to rheumatism in high altitude area

**Figure 8:**
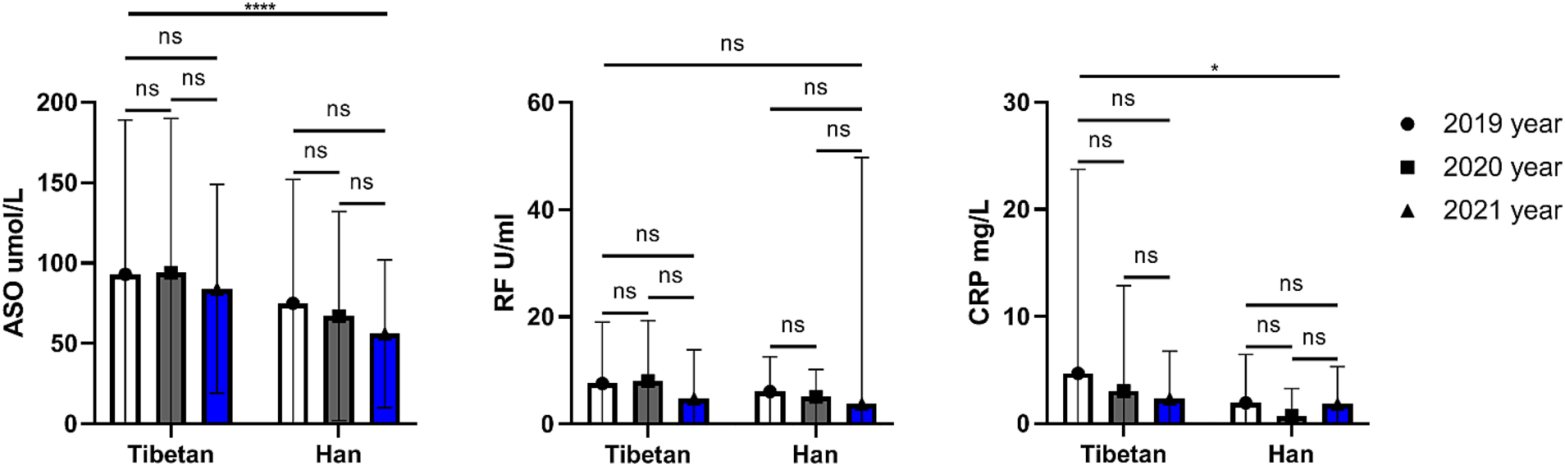
Comparison of rheumatism related indexes in Tibetan and Han. Reference ranges: ASO: 50–150 μmol/L, RF: 0–20 U/ml, CRP: 0–5 mg/L. *p < 0.05, **p < 0.01, ***p < 0.001. Analysis of variance was used for comparison between Tibetan and Han, and paired Student’s *t*-test was used for comparison within groups. *p* <  0.05 was considered statistically significant.

As shown in the Figure 8, the ASO and CRP values of Han people were significantly lower than Tibetan people for 3 consecutive years, and the differences were statistically significant (ASO, *F* = 15.17, *p* < 0.001; CRP, *F* = 5.681, *p* = 0.018). The RF value of Tibetan was higher than that of Han people for 3 consecutive years, but the difference was not statistical significance.

## Discussion

In this study, the data shown there are significant differences in blood and biochemical levels between Tibetan and Han populations who have lived in extremely high altitudes for a long time. Changes in routine and biochemical indicators in blood have been proven to be related to various physiological and pathological processes in the human body, such as inflammation, tumors, heart disease, etc. (24–27). Our study is the first to review the changes in hematological parameters in a low-altitude population (Han) over a period of 3 years after long-term migration to extremely high altitudes for work or other reasons, and compare them with those who have been accustomed to high altitudes for generations Legend has it that the extremely high altitude (Tibetan). After 3 years of continuous observation, we found that platelet, white blood cells and red blood cells of the Han people who migrated to extremely high altitudes all increased, and there were differences to varying degrees from the local Tibetans. This shows that the hematopoietic system has been in a state of metabolic stress in order to adapt to the environment and maintain health, and this stress state has not been relieved over time. Most studies in this area are cross-sectional and assess individual indicators (28–31).

Red blood cells contain a large amount of HGB, which not only acts as a blood buffer, but also transports oxygen and carbon dioxide, and is also an important criterion for clinically judging anemia (32, 33). In this study, the RBC, HGB, HCT, and MCHC values of the Han population in extremely high altitude areas were significantly higher than those of the local Tibetan population for 3 consecutive years. The average level of red blood cells in the Han nationality was higher than the upper limit of the reference range and close to the critical level of high altitude polycythemia (HAPC). HGB levels were also similar to RBC, suggesting that even in Han Chinese who migrated to extremely high altitudes for a long period of time, their HGB and RBC counts remained compensatoryly high to maintain oxygenation of body tissues. Studies have shown that the average blood oxygen saturation of Han people after migrating to the plateau is between 87 and 93%, and they often need oxygen inhalation to maintain blood oxygen saturation (34). Like the Tibetan population, the MCHC value in the blood of the Han population also showed an increasing trend year by year for three consecutive years, and there was a statistical difference year by year. This shows that in order to adapt to the harsh environment, human beings are changing their compensation mechanism to better adapt to life at extremely high altitudes, but Tibetans who have lived for a long time are better able to adapt to the environment and climate. Interestingly, we observed that the RDW values decreased year by year in the Tibetan and Han populations, the difference was statistically significant and within the normal range. The author speculates that it may be because under pressure, the body exerts the maximum potential of bone marrow hematopoiesis, and red blood cells are more efficient and uniform, which is conducive to carrying oxygen. Studies have shown that gender and age have no effect on changes in high-altitude red blood cell levels, while the effect of race is more pronounced (35).

We can clearly observe that the number of platelets is also gradually increasing, and the gradual increase of platelets within the race is not obvious and the difference is not significant, which is consistent with the research results of some recent studies (36–38). However, some scholars have reviewed the changes of platelets in the population at high altitudes through meta-analysis, and the observed results are contrary to the results of this study. They concluded that chronic hypoxia leads to decreased platelet count and increased mean platelet volume (39). Previous studies have shown that excessive compensatory proliferation of erythrocytes leads to decreased platelet counts under hypoxic conditions (40–42). The proliferation and differentiation of megakaryocytes in the bone marrow under stress conditions may increase the number of platelets (43). Other studies have suggested that thrombopoietin may play a complex role in regulating hypoxia and hypobaric exposure (44, 45). However, many literatures currently support the conclusion that platelet reactivity is enhanced under plateau hypoxic environment, and its mechanism lies in the enhancement of Ca^2+^ level and calpain activity (46, 47), the change of blood viscosity (48, 49) and endothelial cell damage in hypoxic environments. Therefore, research in this area is controversial and requires further research and discussion.

Compared with the Tibetans, the platelet counts of the Han people were still slightly lower, the difference was statistically significant, but it was at the higher end of the reference range. At the same time, we also found that the blood PDW and MPV values of the study population decreased with age, but were still within the normal range. The exact reason is unknown, but we believe that this difference may be related to ethnicity, dietary habits and environmental stress, and further research is needed. There are few comparative studies in this area. However, epidemiological data show that the risk of stroke and venous thrombosis increases 30-fold in long-term hypoxic environments above 3,000 meters (50). Furthermore, exposure to high altitudes is associated with an increased risk of thrombotic events and various thrombotic disorders (51). The authors propose that relatively high platelet counts may increase the risk of thrombosis in people at high altitudes.

The data for three consecutive years show that the total number of leukocytes in the emigrated Han population is significantly higher than that in the Tibetan population, and the absolute number and proportion of lymphocytes and monocytes have increased significantly. This indicates that there is a higher immune stress in the Han population and there are racial differences. The authors analyzed that the increase of lymphocytes and monocytes in this study may be due to hypoxic immune adaptation (hypoxia sensitivity of lymphocyte membrane K channel genes) (52). Both hypoxia and hypoxia have effects on the proliferation, differentiation and apoptosis of immune cells. Clinical manifestations include splenomegaly and thymic atrophy, which can severely affect the metabolism of immune cells (53). In addition, it can also regulate the expression of related genes, enhance intracellular glycolysis, affect the energy supply of cells, and ultimately lead to decreased immunity (54). In the past 3 years, no obvious changes were seen in the white blood cell lines of the two ethnic groups. This suggests that Tibetan and Han populations have similar immune stress when adapting to very high altitude environments, but Tibetan populations may be more adapted to very high altitude environments with lower immune stress. This suggests that Tibetan and Han populations may have differences in immune stress when adapting to extremely high altitude environments, and Tibetan population may be more adapted to extremely high altitude environments, but show lower immune stress. Similar studies have shown changes in the leukocyte lineage (55).

Cholesterol is a double-edged sword for the body. When it is too high, atherosclerosis, coronary heart disease and hypertension are prone to occur, and diet and drug intervention are needed. Too low will affect the stability of the cell membrane, the synthesis of sex hormones, and the synthesis of vitamin D (56). This study found no significant change in total cholesterol between Tibetan and Han immigrants. Although there are differences in the comparison of different years within different ethnic groups, the author believes that it may be caused by the deviation of the detection itself, and there is no uniform upward or downward trend. Interestingly, in the 3-year data, we found that the TG value of the Han population was significantly higher than that of the Tibetan population, and the TG value was slightly higher than the upper limit of the reference range. This suggests that the cold and lack of oxygen may cause the body to store more fat in response to high energy expenditure. Dietary changes may also increase intake of exogenous fats, leading to elevated triglycerides. But these factors may increase the risk of cardiovascular disease among immigrant Han Chinese.

The good news is that blood HDL levels in all study populations were within the normal reference range, with an average of more than 1.3 millimoles per liter. In addition, the HDL level of the Tibetan population was slightly higher than that of the Han population, and the difference was statistically significant. Elevated HDL is known to reduce the risk of atherosclerosis and cardiovascular disease, and elevated HDL is associated with physical activity as well as high-quality protein intake (57). The region’s main diet consists of beef and lamb, dairy products and barley, which are rich in high-quality protein. In addition, due to genetic reasons, Tibetans have high blood oxygen content, so they often exercise at high altitudes. This may also be the reason for the high index, of course, further research is needed.

We found that the blood urea level of the Han population was higher than that of the Tibetan population for 3 consecutive years, indicating that the protein metabolism of the Han population at extremely high altitudes is at a higher level and requires better kidney function to support body balance. Serum creatinine is closely related to the total amount of muscle in the body related and less susceptible to diet (58). Creatinine is a small molecular substance that can be filtered through the glomerulus and is rarely absorbed in the renal tubules. Almost all creatinine produced by the human body is excreted with urine every day, and is generally not affected by urine output (59). The results of this study show that the serum creatinine value of the Han population is higher than that of the Tibetan population, and the difference is statistically significant, suggesting that the muscle loss rate of the healthy Han population may be faster at extremely high altitudes.

Hyperuricemia has always plagued people in high-altitude areas, including various ethnic groups, especially in extremely high-altitude areas, where most individuals have uric acid metabolism problems (60, 61). According to the latest data from the Tibet Autonomous Region Center for Disease Control and Prevention, the prevalence of hyperuricemia in residents aged ≥18 is 16.86%, which is much higher than the average level in China (61). In this study, the mean serum uric acid levels of both Tibetan and Han settlers were higher than the upper limit of the normal reference range. The data for three consecutive years show that the blood uric acid level of the outbound Han people is significantly higher than that of the Tibetans. The multi-organ disease and joint problems caused by long-term high uric acid are very obvious in high altitude areas (62). Different from our study, the author surveyed more than 10,000 people on the Qinghai-Tibet Plateau and found that gender, race and altitude are the main factors affecting the blood uric acid level and high uric acid detection rate of healthy adults in this area, among which Tibetans are significantly It is higher than other ethnic groups and tends to increase with altitude. The authors suggest that this may be related to ethnic differences in dietary habits, including sugar, protein and alcohol intake, which requires further investigation.

The results showed that blood levels of LDH and α-HBDH in Tibetan and Han migrants decreased for three consecutive years, but there was no significant difference between ethnic groups. Compared with the Tibetans, the CKMB in the blood of the immigrated Han people was slightly increased, suggesting the possibility of hemorrhage, acute myocardial infarction and other malignant diseases (63), but it has no clinical significance. Therefore, for healthy people, the indicators used to evaluate the myocardial state are not significantly different from those of genetic and transplanted people, and it may be necessary to introduce other indicators for long-term observation.

ASO is commonly used to detect rheumatic diseases. ASO is elevated when rheumatic disease is present, but a high ASO may be the result of a previous infection with hemolytic streptococci. Interestingly, this study found that ASO in the blood of Tibetans was significantly higher than that of Han Chinese in three consecutive years of testing. This may also be one of the reasons why Tibetans are more prone to arthritis, which requires further data support. CRP is not only a non-specific marker of inflammation, but also directly participates in cardiovascular diseases such as inflammation and atherosclerosis, and is the strongest predictor and risk factor for cardiovascular diseases. Interestingly, the study found that blood levels of CRP were significantly higher in Tibetans than Han Chinese. Some scholars have found that CRP is higher in patients with mental failure at high altitude in Han nationality. Whereas our findings were quite the opposite, considering that healthy people did not have such significant effects like inflammation. In addition, it is necessary to be alert to the abnormality of this index caused by occult cold abscess (64) (tuberculosis).

Three consecutive years of research have found that the content of AST in the blood of Tibetans at extremely high altitudes is significantly higher than that of Han Chinese. Although AST/ALT is not greater than 1, it may have a certain impact on liver function. We also found that in three consecutive years of testing, the GGT level in the blood of Tibetans was slightly higher than that of Hans, and the difference was statistically significant. In healthy people, some lifestyle habits, such as alcohol consumption, may lead to elevated GGT (65). Although there is no direct survey data, the results of this study seem to explain this fact indirectly, and further investigation is needed. In this study sequence, the level of alkaline phosphatase in the blood of the Tibetan population was significantly higher than that of the Han population. Its average value is between 90 and 100 U/L. The authors believe that the first reason may be that the Tibetan diet is rich in fat, which may lead to elevated ALP. In addition, the high level of ALP also indirectly reflects the relatively active metabolism of the Tibetan population. It is necessary to further test the level of its isoenzymes to further understand the source. After three consecutive years of comparison, it was found that the blood albumin of the Han population was significantly higher than that of the Tibetan population, and the average value was near the upper limit of the reference range. The author thinks that there may be two reasons. One is that blood concentrations at very high altitudes lead to spurious increases in albumin concentrations. On the other hand, it may be due to changes in the diet structure of the Han people and the increase in foods with high nutritional value.

We also found that the blood levels of TBIL and DBIL in the Han population were significantly higher than those in the Tibetan population for 3 consecutive years. Even the average level of TBIL in the Han nationality has exceeded the upper limit of the normal reference value for three consecutive years. The mean value of DBIL was also near the upper limit of the normal reference range. This shows that the metabolic burden of the liver is heavier in the Han population at extremely high altitudes.

## Conclusion

In summary, there are significant differences in blood and biochemical levels between Tibetan and Han populations who have lived in extremely high altitudes for a long time. While there are still some limitations in this study, firstly, this is an retrospective cohort study, secondly, factors such as weight change were not included in this study. Future study still needed so as to have a better interpretation of this conclusion.

## Data availability statement

The data analyzed in this study is subject to the following licenses/restrictions: the data that support the findings of this study are available from the corresponding author upon reasonable request. Requests to access these datasets should be directed to CC, chencheng@xjtu.edu.cn.

## Ethics statement

The humans and animal study were approved by Ethics Committee of Ali District People’s Hospital. The studies were conducted in accordance with the local legislation and institutional requirements. The human samples used in this study were acquired from primarily isolated as part of your previous study for which ethical approval was obtained. Written informed consent for participation was not required from the participants or the participants’ legal guardians/next of kin in accordance with the national legislation and institutional requirements.

## Author contributions

ZY: Data curation, Methodology, Project administration, Writing – original draft. YZ: Data curation, Writing – original draft. XL: Data curation, Writing – original draft. LW: Conceptualization, Data curation, Methodology, Writing – review & editing. CC: Conceptualization, Supervision, Writing – review & editing.
